# Understanding the role of grievance and fixation in lone actor violence

**DOI:** 10.3389/fpsyg.2022.1045694

**Published:** 2022-12-22

**Authors:** Nathan Brooks, Justin Barry-Walsh

**Affiliations:** ^1^College of Psychology, School of Health, Medical and Applied Sciences, Central Queensland University, Townsville, QLD, Australia; ^2^Behavioural Science Unit, New Zealand Police, Auckland, New Zealand; ^3^Fixated Threat Assessment Centre, Wellington, New Zealand; ^4^Te Whatu Ora Health, Capital Coast, Hutt Valley and Wairarapa, Wellington, New Zealand

**Keywords:** grievance, fixation, violence, lone actor, terrorism, extremism

## Abstract

In pursuit of public discourse, there is a risk of a simple polarity in thinking, meaning acts of public/mass violence, or those where there is a risk of public violence are categorized as terrorist acts or not. The reliance on categorization, and the pursuit of assigning ideology, diminishes the complexity of factors contributing to these forms of offending. This approach misses a critical opportunity to understand the pathways to violence and reduces the significance of comorbid or co-occurring factors that give rise to the violence. Lone Actor Grievance Fueled Violence (LAGFV) is a recently utilized term based on mounting evidence that those who seek to perpetrate acts of lone actor violence, whether this be those where a terrorist motivation can be assigned, a school attacks, workplace attack, or an attack in a public place, are commonly fueled by grievance and fixation. LAGFV at this juncture is a blurry construct without definitive rules and boundaries, and instead provides a guiding conceptualization of a diverse group of offenders who commit targeted violence towards others. The current review contends that LAGFV emerges through the perceived thwarting of psychological needs and the central task for clinicians and other professional services lies in understanding the unique pathways and contributing factors that give rise to violence for each specific individual/case. The review contends that is not fit to determine whether an act is of ideological significance, or constituting terrorism, without understanding the psychosocial and circumstantial factors contributing to the violence.

## Introduction

In May 2021, Luke Lambert entered a Countdown supermarket in Dunedin, New Zealand, and removed a packet of four knives from a shelf. He removed two of the knives from their packaging, each with a seven-centimeter blade. He then approached a female staff member of the store, cutting her across the face from the ear lobe to chin. Lambert proceeded to climb on top of her and continued the attack, stating, “*I’m going to kill you*” (Kidd, 2021). A nearby store manager attempted to intervene and pull Lambert off the woman, however, in the process he was stabbed three times. Lambert then attacked a husband and wife who were frequenting the store. He stabbed the female in the back twice, before, attacking the male. With the couple lying on the ground, Lambert continued to stab the pair, before being restrained by supermarket staff, members of the public, and off-duty police. In the hour prior to the attack, Lambert had visited the store, walking around for 30 min before purchasing a pie and a soda drink. He also attempted to purchase two cans of beer; however, his bank card was declined. He was reportedly agitated following this incident, and commented to an associate afterwards, “*someone is going to get it*” (Lloyd, 2021). A short time later he returned to the supermarket store and carried out the attack; fortunately, all four victims survived.

In early September 2021, Ahamed Aathil Mohamed Samsudeen entered a countdown supermarket in Auckland, New Zealand, and removed a knife from the shelf. He proceeded to attack members of the public shopping inside the store, stabbing seven people with the knife. At the time, Samsudeen was under 24-h surveillance by police, and within minutes of the attack commencing, police shot him dead in the store. Although four victims were initially in a critical condition, all victims survived the attack. Samsudeen was a Sri Lankan national who had arrived in New Zealand in 2011, however, in 2016, he was formally warned by police about posting objectionable material on the internet and expressing his support for Islamic State terrorist activities. In 2017, he was arrested at Auckland airport after having purchased a one-way ticket to Malaysia. He had confided in an associate about wanting to travel to Syria to fight for the Islamic State. A search of his apartment by police uncovered a large hunting knife underneath his mattress, an array of digital fundamentalist material, a photograph of him posing with a firearm, and bookmarked websites to sales of military boots, a vest, binoculars, crossbows, and firearms (Robson, 2021). He was charged by police and remanded in custody, and after 13 months of remand, he was released on bail in June 2018. Shortly after his release, Samsudeen searched the internet for camouflage trousers and purchased another hunting knife in August 2018. He was again arrested, and police discovered he had been accessing further Islamic State material on the internet. Whilst in custody, Samsudeen received further charges after attacking two correctional officers. In May 2021, he was sentenced on two charges of possessing Islamic State propaganda and one charge of failing to comply with a search. After spending 3 years in custody, Samsudeen was released from prison in July 2021. Police commenced 24-h surveillance of Samsudeen due to concerns over the likelihood that he may perpetrate an attack. Approximately 3 months later, Samsudeen carried out his attack at the Countdown supermarket (Bonnett, 2022).

The attacks perpetrated by Lambert and Samsudeen are concerning due to the ease in which these acts were perpetrated, along with the striking similarities in the methods employed. Speculation may presume that Samsudeen’s attack, which may or may not have been planned, was substantially influenced by the actions of Lambert. However, Lambert’s attack was considered ‘random’ and therefore not constituting an act of domestic terrorism (RNZ, 2021). On the other hand, within hours of Samsudeen’s attack, government officials had characterized his offending as terrorism, stating Samsudeen was an “*ideologically motivated violent extremist*” and a “*terrorist*” (New Zealand Parliament, 2021). Currently, the motivations of Lambert remain unclear and there is no evidence of any ideologically inspired violence. He had told an assessing probation officer that the death of his mother in 2018 and the subsequent deaths of his dogs was traumatic and a significant turning point in his life. Following these events, Lambert’s struggled financially and ended up living in his car. However, after crashing his car, he had no reliable shelter and became homeless. He spent much of his available money on alcohol and engaged in cannabis and mushroom use (Lambert, 2022). His lawyer stated that Lambert was suffering from severe mental health issues at the time of the incident, however, mental health assessments determined that he was not psychotic, nor of unsound mind at the time of the offence (Kidd, 2022). A previous assessment of Lambert identified that he had “*an usual and eccentric personality structure rather than…an underlying functional psychotic illness such as schizophrenia or delusional disorder*” although an underlying psychotic illness could not be completely excluded (Lambert, 2022, p. 10).

For Samsudeen, an extensive history of Islamic State support was evident, along with a disdain for western society. He arrived in New Zealand having been subjected to trauma in Sri Lanka and failed to find acceptance and a new sense of identity after immigrating (Buchanan, 2021). He was reportedly lonely and had limited social connections, with episodes of depression and anger towards what he perceived as an unjust world. When assessed by a psychologist, Samsudeen was noted to have the presence of “*multiple mood disorders*” and considered “*a highly distressed and damaged young man*” (Manch, 2021). His psychosocial difficulties were further compounded by his reluctance to engage with rehabilitative efforts, and steps to revoke his visa by the government, further worsened his attitude and willingness to work with authorities.

The actions of Lambert and Samsudeen were classified as ‘not terrorism’ and ‘terrorism’. However, in pursuit of public discourse, there is a risk of a simple polarity in thinking, meaning acts of public violence (or those where there may be risk of public violence), are categorized as terrorist acts or not. This creates a narrow focus on those thought to have potential terrorist motivations, often based on simplistic assumptions about pathways to radicalization and violent extremism. For instance, Samsudeen “*had no manifesto and he did not video his actions or communicate or transmit his attack to others. He had no subject other than his immediate targets and he had no object than to satisfy his own bloodlust and sense of being wronged by society…He had no connections to any jihadist network…although he glorified ISIS violence and fetishized bladed weapons, it is unclear how deeply rooted he was in the Salafist world view that underpins ISIS’s ideology*” (Buchanan, 2021, para. 11–12). Consequently, the reliance on categorization, diminishes the complexity of factors contributing to these forms of offending. Moreover, this approach misses a critical opportunity to understand the pathways to violence, and reduces the significance of comorbid or co-occurring factors that give rise to the harm that occurs when someone perpetrates this form of violence. There are number of problems with the reductionist terrorist/not terrorist or radicalized/not radicalized dichotomy. As observed by Corner et al. (2018), “…*solely focusing on those who engage in violence on behalf of a political or religious cause unduly narrows our understanding of the relationship between mental disorder and personality traits and extreme violence*” (p. 461).This position does not seek to detract from the critical importance of knowing and understanding about pathways to radicalization, extremism and terrorism, but instead to acknowledge that this form of offending is nuanced, marked by complexity and a myriad of co-occurring contributing factors. Due this, the authors propose that the acts of Lambert and Samsudeen and other public mass violence, can both be better understood and better prevented by more broadly conceptualising these as acts of Lone Actor Grievance Fueled Violence (LAGFV). While some of those who perpetrate LAGFV will meet criminal code legislation to be charged with a terrorism offence, this does not directly suggest that their offending was intended to be an act of terrorism. Similar parallels can be drawn to offences classified as ‘murder’ under relevant criminal codes (Australian Government, 1995; Parliamentary Counsel Office, 2021). Although an offence of homicide may occur, there are often many different factors and motivations that lead to homicide offending (Hazelwood and Douglas, 1980; Burgess et al., 1986; Malmquist, 2006; Petherick and Petherick, 2018). These are essential to understand conceptually, empirically, and clinically.

LAGFV is a recently utilized term, based on mounting evidence that those that seek to perpetrate acts of lone actor violence share a number of characteristics; whether this be those where a terrorist motivation can be assigned, a school attack, workplace attack, or an attack in public places (Pathé et al., 2018; Barry-Walsh et al., 2020; Brooks and Shaw, 2022). Further, this conceptualization provides an opportunity for intervention and prevention prior to such attacks through the identification of antecedents and contributing factors to violence (Capellan, 2015; Pathé et al., 2018; Clemmow et al., 2022). LAGFV serves as an umbrella term to conceptualize this broad cohort of offending, with grievance and/or fixation considered universal contributors to lone actor violence. Although this terminology could also be represented as Lone Actor Grievance and Fixated Fueled Violence (LAGFFV), for the purpose of consistency with prior research (Pathé et al., 2018; Barry-Walsh et al., 2020; Brooks and Shaw, 2022; Clemmow et al., 2022; Ebbrecht, 2022), the present review will refer to this offending as LAGFV which is considered to encompass the role of fixation. LAGFV at this juncture is a blurry construct without definitive rules and boundaries, and instead provides a guiding conceptualization of a diverse group of offenders who commit targeted violence towards others. The conceptualization of LAFGV serves to capture a clustering of offences, however, an analysis and formulation of each act/perpetrator is required to specifically understand unique ideologies, motivational factors, mental health concerns, modus operandi, and intent. Although the purpose of the present analysis is to examine LAGFV, in general, grievances may contribute to many forms of targeted violence. This form of violence may encompass targeted offending such as domestic violence, stalking, lone actor and mass casualty events, and those that make threats to harm (Brooks et al., 2021; Corner et al., in press). The central commonality amongst this form of offending is the pursuit of violence towards a specific victim or broader sub-population that emerges due to a perceived experience of injustice, victimization, rejection, or other experiences that may challenge a person’s identity or sense of belonging. Among these various forms of offending is the intention to carry out violence, or to convey the ability to inflict harm to achieve outcomes such as justice, retribution, and/or revenge. While the boundaries and definition of grievance fueled violence remain broad and unrefined, it is the position of the authors that this form of violence relates to acts intended to achieve revenge, retribution, justice, the restoration of natural order, or the righting of a wrong. Although grievance fueled offending is often targeted at a specific victim/group, or something/someone of symbolic significance, this form of violence may also be targeted at an accessible and available proxy victim group (e.g., a lone actor who targets a public crowd due to disenfranchisement and moral outrage and seeks to make others suffer and understand the extent of their plights).

The emergence of LAGFV as a valid concept has occurred on the back of the development of threat assessment as a maturing area of research and practice (Pathé et al., 2018; Corner et al., 2019; Meloy et al., 2019). Threat assessment and management practices have been developed to assist law enforcement, counterterrorism agencies, mental health services, and correctional departments, determine risk and prioritize responses to cases of LAGFV. This has seen the development of Fixated Threat Assessment Centres and their expansion into the LAGFV area (Pathé et al., 2018; Barry-Walsh et al., 2020; Wilson et al., 2021). It has seen the development of tools, still in their empirical infancy, to aid in assessing threat and concern. Some of these tools are the Terrorist Radicalization Assessment Protocol (TRAP-18; Meloy, 2017), the Violent Extremism Risk Assessment-2 Revised (VERA-2R; Pressman and Flockton, 2010; Pressman, 2014), and the Extremist Risk Guidelines-22 (ERG-22+; Lloyd and Dean, 2015).

At a fundamental level, LAGFV perpetrators engage in hostile acts against others to achieve outcomes of a particular meaning to them (Pathé et al., 2018). Compared to group actors, or those that carry out group-based terrorism, lone actors have been found to have higher rates of mental illness (Corner and Gill, 2015; Corner et al., 2018, 2019), more prevalent criminal histories (Gill et al., 2014), an older average age (Gill et al., 2014), a preference for firearm use (Jasparro, 2010; Spaaij, 2012; Gruenewald et al., 2013), and typically target crowded places rather than critical infrastructure or government locations (Spaaij, 2010, 2012; Eby, 2012; Teich, 2013).

LAGFV encompasses a broad range of offending that is perpetrated generally by one person, although occasionally perpetrators work in dyads or triads, finding connection with others who share their violent interests, and together furtively plan and conduct an attack ( De Roy et al., 2016; Turner et al., 2021). The use of this umbrella term serves as an overarching conceptualization for individuals who seek to carry out targeted violence against others for political, social, or personal reasons. From both a construct and clinical perspective, broad categorization “…*provides a means of organizing pathological phenomena, the signs, traits, and symptoms (manifestations) of personology and psychopathology*” (Meehl, 2011, p. 235). Consequently, LAGFV captures a breadth of offending, with some cases motivated by far-right, far-left, or jihadist inspired ideologies, and constituting terrorism (Silva, 2022). Other cases may result from a mix of mental illness, personality pathology, criminality, or complex clinical histories, therefore, blurring the line on motivation, intent, and ideology (Corner and Gill, 2015; Pathé et al., 2018; Winter and Spaaij, 2018; Barry-Walsh et al., 2020). The current paper contends that LAGFV serves as a guiding conceptualization for a range of offending and perpetrators and the central task for clinicians and other professional services lies in understanding the unique pathways and contributing factors that give rise to violence for each specific individual/case. As discussed, the reliance or whether terrorism or not (particularly at a clinical or operational level), diminishes the complexity of factors contributing to LAGFV offending. Moreover, a significant emphasis on ideologies is often misplaced, given instances of shifting ideologies, piecemeal and fragmented beliefs, and muddied motivations (Miller-Idriss and Hughes, 2021). The paper will examine the utility of an across-the-board view of LAGFV, with an emphasis placed upon determining the motivational influences and clinical building blocks pertinent to each individual. It is the position of the authors that it is not suitable to determine whether a terrorist motivation can be assigned to an offender, nor whether an offence is ideologically inspired, without understanding the psychosocial and circumstantial factors which have precipitated the violence.

## Motivational influences and the role of grievance and fixation

There is no single accepted definition of what constitutes terrorism (Nacos, 2019). Many countries or states have a criminal code or offence definition for charging and convicting offences of terrorism. For instance, both Australia and New Zealand define a terrorist acts as one that is done to advance a political, religious or ideological cause, typically resulting in death, endangerment to the public, or causing a serious risk to health and safety (Parliamentary Counsel Office, 2002; Australian Government, 2021). Although criminal code legislation stipulates conditions for an offence, this does not directly indicate an established definition of terrorism. Moreover, as Gill (2015) discussed, terrorism has for many decades been viewed through a group lens rather than at an individual level. This lack of consensus, while important to grasp., does not necessarily impede the examination or research in the field, with a range of conceptual, theoretical and empirical research and analysis possible (Schuurman, 2020). Indeed, contingent on the context and purpose, variation in definition may be desirable (Bouhana, 2019). In light of this, and because of the authors’ position on dichotomous categorization, the present review will not advance a definition of terrorism. Regardless of whether an offence constitutes terrorism or not, there are a variety of constructs and domains which may influence a person’s motivation and pathways to offending.

Motivations for violence are complex and nuanced, and as Jacobs and Wright (1999) observe, “*motivation is criminology’s dirty little secret - manifest yet murky, presupposed but elusive, everywhere and nowhere*” (p. 149). While grievance and/or fixation typically serve as universal contributors to lone actor violence, many perpetrators are influenced by ideological identifications. The most commonly identified ideologies include, left-wing, right-wing, or Jihad-inspired ideologies (Silva, 2022). However, offending can rarely be classified into a single motivational or ideological category. Instead, a variety of ideologies, motivations, or beliefs, may contribute to offending behavior. As observed by Winter and Spaaij (2018) “*The motivations for lone actor attacks are often on the blurry line…driven by an intermingling of personal, political and social factors*” (para. 9). In recognition of this, Australian Security Intelligence Organisation (2021), has moved away from the use of discrete labels to classify offending motivations, noting that these terminologies are no longer suitable for understanding lone actor violence.

The annual report by Australian Security Intelligence Organisation (2021) stated, “*Terms like ‘left-wing extremism’ and ‘right-wing extremism’ are no longer fit for purpose… Instead, we now use religiously motivated violent extremism and ideologically motivated violent extremism as umbrella terms, with more specific terminology available when we refer to particular threats*” (p. 5). However, by classifying offending based upon a religious or ideological dichotomy, there is a risk that this promotes a narrow view of offending, failing to understand the underlying factors that give rise to such violence. In simplest terms, ‘a cake is not the product of its icing’. Similar categorization has been employed in other countries such as Canada and New Zealand. For instance, New Zealand has identified four categories of ideological motivations (identity-motivated violent extremism, politically-motivated violent extremism, faith-motivated violent extremism, and single issue-motivated violent extremism) to classify offending (Combined Threat Assessment Group, 2022). These attempts to classify behavior or ideologies have utility and in many ways are comparable to the body of research and literature that has classified sexual, violent, and serial forms of offending into typologies to distinguish various motivations and forms of offending behavior (Hazelwood and Douglas, 1980; Douglas et al., 1986; Holmes and Holmes, 2002; Canter et al., 2004). However, it is often the underlying psychosocial processes that make a person susceptible to these influences. Moreover, movement towards causes or ideologies, typically occurs during periods of significant stress, loss, disenfranchisement, and/or injustice (Gill, 2015; Pathé et al., 2018). It is at this stage where grievance and/or fixation may emerge and serve to precipitate mobilization towards violence.

According to Pathé et al. (2018), “*Lone-actors engage in hostile acts against others in pursuit of aims that have a particular meaning for them. Their violence is underpinned by a sense of injustice, loss, injury or victimization…Typically, all harbor some personal grievance or vendetta, triggered by a perceived injustice*” (p. 38–39). Alongside this, many of those harboring grievances “*spend much of their waking lives thinking about the object of their concern. They usually gather information from multiple sources, including newspapers, books, television, and, increasingly, the Internet*” (Mullen et al., 1999, p. 34). An examination of active shooter incidents within the United States revealed that 79% of perpetrators acted based upon some form of grievance, with 49% of these grievances relating to interpersonal circumstances or perceived employment wrongdoings (Silver et al., 2018). In addition to this, perpetrators on average experienced 3.6 life stressors in the year prior to committing the attack. Further examination of mass violence and lone actor attacks indicated that 81% of the 377 perpetrators analyzed displayed evidence of pathological fixation (Meloy and Rahman, 2021).

Grievances may be political, social, personal, or due to an entanglement of many factors. The attack perpetrated by Elliot Rodgers at sorority house at the University of California in May 2014 was fueled by misogynistic beliefs, blame towards women, and a sense of injustice and humiliation (Meloy et al., 2019; Ebbrecht, 2022). The Unabomber, Theodore Kaczynski, targeted industries, and individuals, whom he perceived where affecting the environment and enslaving people to a life governed by technology (Allely, 2020). Andrew Joseph Stack III flew his small Piper Dakota plane into the IRS field office in Austin, Texas, following a series of taxation issues, a marriage breakdown, and having his company operations suspended. The actions of 21-year-old Dylann Roof were fueled by anger and hatred towards African Americans. Roof opened fire on the congregation at the Methodist Episcopal Church in Charleston, South Carolina, believing that “Blacks were taking over the world” (Silverstein, 2015). Lastly, in New Zealand, the offending of Luke Lambert emerged from a sense of hopeless, hardship, and disenfranchisement with society. Lambert’s general disfranchisement with society, coupled with his psychological vulnerabilities, contributed to him blaming and perceiving others as being responsible for his hardships and personal difficulties. In contrast, Samsudeen harbored anger at the West, supported an Islamic values structure, felt victimized and targeted, and had an unresolved history of trauma.

According to Ebbrecht (2022) grievances result from unmet or thwarted psychological needs, with violence used to seek revenge, retribution, or to right a wrong. This can occur when a person experiences a threat or challenge to their identity, sense of belonging, areas in which they derive meaning and significance, or their independence, liberty, and wellbeing. This may arise through humiliation, the sense of being shamed, the experience of prejudice, injustice, rejection, discrimination, ostracism, harassment, bullying, victimization, inequality, or a traumatic event (Borum, 2015; Winter and Tschudi, 2015; White, 2017). Although the severity or nature of these circumstances or even can vary, it is the subjective experience and perception of the individual that is paramount to the development of a grievance. This can be further compounded by the presence of mental illness, most obviously through psychosis or personality psychopathology. It is noted that “*motives always reflect a need, that is, an intrapsychic state of tension due to a psychological lack or deficit…the motive, in turn, directs behavior towards need satisfaction*,” suggesting grievance-fueled violence functions as a means of psychological need restoration (Ebbrecht, 2022, p. 24).

Although grievances can develop through various threats and challenges to psychological needs, many people can experience grievances without progressing to violence. The experience of a thwarting to a psychological need, may affect individuals differently and result in different responses and outcomes. In one person, a situational circumstance may serve to exacerbate a long-term grievance, while for another, a pre-existing capability for violence may be precipitated by an event or incident that results in the development of a grievance and fosters an intent to act (Corner and Taylor, in press). The differences in individual responses, provides further evidence that viewing lone actor violence through a lens of labels and classifications overlooks the precursors and psychosocial building blocks that lead a person to violence. Moreover, by understanding the broader role of grievance and fixation in contributing to lone actor violence, it is possible to examine the range of factors that contribute to the development of this psychological state.

## Understanding the build blocks

In understanding LAGFV, it is important to consider two essential questions about the development of grievance-fueled violence (Wolfowicz et al., 2021) Firstly, why do some individuals who experience threats or challenges to their psychological needs develop grievances when most of those who experience similar circumstances or events do not? Secondly, why is it that some people who develop a grievance resort to violence while many continue living their life?

The challenges of understanding the pathways to grievance-fueled violence shares similarities with the early literature and research on aggression and violence. In his seminal work, Megargee (1976) described the interactive effects of variables in giving rise to aggressive violence. Rather than focusing on individual factors as causes, Megargee (1982, 1993, 2002, 2009) recognized the complex interaction between personal, situational, and inhibitive factors in violence. He termed this phenomenon the “Algebra of Aggression”. The Algebra of Aggression acts as conceptual framework for understanding all forms of human aggression, focusing on factors and their interactive relationship in the formation of aggressive responses (Megargee, 2011). The construct of LAGFV is still in its infancy, and although grievances have been a commonly cited motivation for violent behavior across cases and research (Mullen et al., 1999; Gannon et al., 2012; McEwan et al., 2017; Corner et al., 2018; Brooks et al., 2021; Brooks and Shaw, 2022; Douglas, 2022), there is a dearth of conceptual and theoretical understanding pertaining to grievance-fueled violence (Corner and Taylor, in press). Consequently, it is imperative to understand the ‘Algebra of Grievance’, or more specifically the primary contributing factors and their interactive effects in grievance-fueled violence.

According to Borum (2011, 2015), violent extremism arises from a compilation of psychosocial processes and personal and situational circumstances that push a person towards violence. A wide range of features may necessitate the shift from contemplation to mobilization. The complex interaction between these features/factors is acknowledged by Corner et al. (2019) when discussing the role of mental health and criminality alone, noting “*Anger appears to be an important precipitating factor in both the subsequent cyclical violent and criminal behaviors, and religious conversion, while mental health problems appear to be a precursor to, and consequence of, criminal behaviors, which are themselves markers of lack of commitment to prosocial moral rules (moral susceptibility) and/or markers of selection into criminogenic settings, some of which may be radicalising (including prison)*” (p. 120).

The role of grievance in fuelling violence presents the classic ‘chicken or egg’ causality dilemma, with grievance at times leading to maladaptive psychosocial functioning, and vice versa. Borum (2015) suggests that grievances ‘push’ people towards violence and incentives, ideologies, and beliefs, ‘pull’ them towards action. Interestingly, in a recent analysis Corner and Taylor (in press) found that grievance-fueled violence was preceded by instability in living conditions. The experience of instability was exacerbated by social rejection, which in turn was followed by the expression of prejudice and anger towards others along with preoccupation and rumination on thoughts and/or beliefs. At this stage, if a desire to take revenge developed then grievance-fueled violence ensued (Corner et al., in press). The research by Corner and Taylor highlights how life circumstances, social experiences, psychological and emotional states, coupled with a desire for revenge or retribution can foster grievance-fueled violence.

The experience of a disruption or threat towards a psychological need can result in shifts and changes to a person’s cognitive state and mindset. This in turn, can cause changes in emotional states and behavior. The harboring of a grievance alters the lens through which a person perceives the world. This subsequently changes the person’s perception and interpretation of situations and events. A bias and distortion in cognitive processes forms and overtime this leads to changes in a person’s beliefs and attitudes. Consequently, information supporting grievance-based beliefs and attitudes is preferenced, whilst contradictory information is minimized and/or ignored. This type of cognitive state is typically characterized by the following beliefs and attitudes (Little et al., 2021; Meloy and Rahman, 2021; Wolfowicz et al., 2021), these include:

- The rights to freedom and independence, or the rights to fairness and equality
- Equality, or inequality, being laws of nature which should be followed
- The perception of an imminent or existential threat
- A “with us or against us” or “good or evil” perception of people and events
- Rigid and fixed views of right and wrong
- That violence or force is necessary to prevail
- The perception of being chosen, or called upon to act

Maladaptive beliefs and attitudes play an important role in the continuation of a grievance. However, as discussed at the beginning on this section, many people who develop a grievance do not resort to violence as a means of retribution or revenge. Why some people develop grievances and harbor these to the point of enacting violence remains a complex question. In his work on aggression, Megargee (1976, 2011) identified that some people are easily angered, yet, have learnt through social processes and lessons that it is more acceptable to become verbally rather than physically aggressive. Others have been rewarded for aggressive behavior and/or observed peers or family members resolving problems or conflicts through aggression. Everyone experiences anger, yet, most do not resort to acting out in a physically aggressive manner, or more concerningly, using instrumental or calculated aggression. Although it is not as straightforward as comparing the body of research on aggression to the area of grievance-fueled violence, the lessons on the interactive effects of factors in contributing to aggression assist in drawing some parallel conclusions. LAGFV does not occur due to a singular contributing factor such as mental illness, or because of an identification with a particularly extremist ideology. Instead, “*key antecedents that, when in combination, interact with each other over time, resulting in the development of a grievance that fuels an act of violence…the interaction between variables may be of more predictive value than the presence or absence of the variable, and that these interactions over time can produce different pathways for people as they develop a grievance and move towards committing an act of violence*.” (Corner and Taylor, in press).

Although the combination, or threshold for these contributing factors remains elusive, it is the interactive effect that results in violence. While it remains an ongoing challenge for researchers and clinicians to understand why some people harbor grievances and progress to violence, the present hypotheses suggests the response to a perceived grievance or thwarting of psychological needs is mediated by a person’s affect/emotions, habits/actions, beliefs and attitudes, cognitive mindset, social attributes and functioning, personality, mental illness, identity/self-concept, substance use, experience of adverse events or trauma, life circumstances, and personal capabilities.

## Moving towards a clinical perspective

The heart of the issue for professionals and practitioners, is to formulate an understanding “*about how specific internal or situational factors caused or influenced an individual’s decisions or behavior, including interactions with vulnerabilities, and how the array of influencing factors might have affected each other*” (Borum, 2015, p. 77). Although it may be tempting to classify offending as an act of terrorism, or to employ discrete labels to classify acts, such an approach overlooks the complexity of LAGFV. At a criminal charge and conviction level an offence may be classified as terrorism, however, valuable understanding of the offender and their motivations may be lost clinically, conceptually, and empirically if viewed solely through this lens. By using this dichotomous approach to classify offending, pertinent case information pertaining to prevention, risk, or ‘warning behaviors’ may be lost.

In an article published following Ahamed Aathil Mohamed Samsudeen’s supermarket attack in September 2021, Casinader (2021) examined his progression to violence. Several years before his attack, a psychologist identified that Samsudeen was highly distressed, suffering from PTSD and depression, experiencing violent thoughts and socially isolated. Samsudeen’s defence barrister, Aarif Rasheed, argued that he was treated as a terrorist early into his offending. He alleged that Samsudeen was labelled as a terrorist, and he eventually came to perceive himself this way. Although debate will continue about decisions that were or were not made about Samsudeen, it is evident he arrived in New Zealand after having suffered trauma and experienced difficulties with adjusting to the culture and finding acceptance and belonging. Samsudeen struggled with western culture and overtime he became increasingly frustrated with his circumstances. Along with his existing mental health issues, he was angry and prone to blaming others. He began to become disenfranchised with western society and found interest in online forums and platforms which promoted Islamic extremism. His subsequent convictions and periods of imprisonment served to only fuel his grievances and anger. It was this cycle of grievance and unresolved mental health issues that fueled his violence, with various circumstances and events acting as catalysts for action.

Although some may argue that a movement towards LAGFV in lieu of using terms such as ‘terrorism’ or ‘ideology’ is a matter of semantics or an emotional reaction to the aftermath of recent violent attacks across a spate of countries, the position of the authors is that this shift in conceptualization is anything but that. While the actions of perpetrators such as Samsudeen and Lambert do create existential distress and prompt an evaluation of processes, there is ample reasoning to suggest that providing a nuanced understanding of offending benefits communities and those tasked with responding to these tragedies. Moreover, public discourse can be done through the sharing of clinical knowledge and understanding. Without this shift, there also remains the risk that policies or governmental positions may inadvertently shape clinical responses and decision-making pertaining to a person.

The concept of LAGFV and of the burgeoning literature on both Threat Assessment and LAGFV provides an opportunity for prevention and reduction in harm, through a mechanism that allows for earlier recognition of those at risk of such actions. This does not require the technology to predict who will go on to perpetrate LAGFV, rather it allows for identifying distressed and disturbed individuals, often with psychiatric, psychological, educational and social difficulties, and intervening. This not only reduces the risk of their progression to a state where they may act violently, but also reduces the harm and suffering to themselves and those around them. This is what is at the heart of fixated threat assessment services and similar initiatives such as PREVENT (Counter Terrorism Policing, 2022), He Aranga Ake (Department of the Prime Minister and Cabinet, 2021), and Living Safe Together (Australian Government, 2020).

It is important the construct of LAGFV informs clinical and operational decision making. Through identifying the core experiences (such as perceived injustice, discrimination and/or victimization) fuelling a person’s behavior, it is possible to develop a comprehensive understanding of the person and/or the matter. By understanding what is occurring, how a grievance developed, and the subsequent pathways to violence, a more informed and accurate approach to treatment, assessment, and management can occur. If a person’s violence is driven by a grievance, then this becomes a critical risk and responsivity factor that can be examined, measured, and assessed. If alternatively ideological aspects are prioritized, or the person is simply classified as being a terrorist, essential risk and clinical indicators may be overlooked. Instead change may be measured based on a reduction in ideological beliefs, rather than on a shift in underlying cognitions or beliefs relating to injustice, disenfranchisement, or other experiences pertaining to the perceived thwarting of identity or belonging. Without greater adjustment, integration, acceptance, accountability, and progress towards alternative and/or prosocial endeavors, it is likely that the psychological mechanisms giving rise to a grievance will persevere and remain.

With a complex cohort of individuals and offending, clinical expertise is essential to inform policies, practices, and decision-making. The justification for the involvement of health professionals in the area must be to do good, to provide expertise, care, treatment, and advice. Ultimately, there is a need to focus on the individual at risk, as well as the community or people who may be exposed to such risk. In many cases, through collaborative efforts the risk of harm can be mitigated or prevented, benefiting both the person of concern and the broader community. In instances where tragedy and harm occur, a sophisticated understanding and explanation can be provided to explain offending and the individual pathways to violence. This approach to using clinical expertise to understand violence is not a new phenomenon, with psychiatry, psychology and other mental health professionals having a long-standing role in assisting the Courts, the community, and the victims in understanding offending behavior. We contend that the field of targeted and mass violence requires a collaborative and informed shift away from the dichotomy of terrorism/not terrorism. Instead, Lone Actor Grievance Fueled Violence provides an important conceptual framework within which to embed this clinical practice and decision-making. The approach of using clinical expertise to understand violence is not a new phenomenon, with psychiatry, psychology and other mental health professionals having a long-standing role in assisting the Courts, the community, and the victims in understanding offending behavior. Clinical expertise is essential to inform policies, practices, and decision-making. The review contends that the field of targeted and mass violence requires a collaborative and informed shift away from the dichotomy of terrorism/not terrorism. Instead, Lone Actor Grievance Fueled Violence provides (LAGFV) an important conceptual framework within which to embed this clinical practice and decision-making. The paper examines how the concept of LAGFV provides an opportunity for an informed understanding of the clinical and risk factors that give risk to violence. Through examining the role of grievance and other psychosocial factors in lone actor violence, there is an opportunity for improved threat assessment practice and the prevention of harm through the early recognition of clinical and risk indicators.

## Author contributions

All authors listed have made a substantial, direct, and intellectual contribution to the work and approved it for publication.

## Conflict of interest

The authors declare that the research was conducted in the absence of any commercial or financial relationships that could be construed as a potential conflict of interest.

## Publisher’s note

All claims expressed in this article are solely those of the authors and do not necessarily represent those of their affiliated organizations, or those of the publisher, the editors and the reviewers. Any product that may be evaluated in this article, or claim that may be made by its manufacturer, is not guaranteed or endorsed by the publisher.
